# A Novel Wireless and Temperature-Compensated SAW Vibration Sensor

**DOI:** 10.3390/s141120702

**Published:** 2014-11-03

**Authors:** Wen Wang, Xufeng Xue, Yangqing Huang, Xinlu Liu

**Affiliations:** State Key Laboratory of Acoustics, Institute of Acoustic, Chinese Academy of Science, No.21, BeiSiHuan West Road, Beijing 100190, China; E-Mails: xuexufeng@mail.ioa.ac.cn (X.X.); hyq@mail.ioa.ac.cn (Y.H.); liuxinlu1987@foxmail.com (X.L.)

**Keywords:** vibration sensor, surface acoustic wave, Y-cut quartz, wireless and passive, cantilever beam

## Abstract

A novel wireless and passive surface acoustic wave (SAW) based temperature-compensated vibration sensor utilizing a flexible Y-cut quartz cantilever beam with a relatively substantial proof mass and two one-port resonators is developed. One resonator acts as the sensing device adjacent to the clamped end for maximum strain sensitivity, and the other one is used as the reference located on clamped end for temperature compensation for vibration sensor through the differential approach. Vibration directed to the proof mass flex the cantilever, inducing relative changes in the acoustic propagation characteristics of the SAW travelling along the sensing device, and generated output signal varies in frequency as a function of vibration. A theoretical mode using the Rayleigh method was established to determine the optimal dimensions of the cantilever beam. Coupling of Modes (COM) model was used to extract the optimal design parameters of the SAW devices prior to fabrication. The performance of the developed SAW sensor attached to an antenna towards applied vibration was evaluated wirelessly by using the precise vibration table, programmable incubator chamber, and reader unit. High vibration sensitivity of ∼10.4 kHz/g, good temperature stability, and excellent linearity were observed in the wireless measurements.

## 1. Introduction

In recent years, the surface acoustic wave (SAW) devices have gained increasing attraction for wireless sensing for temperature, pressure, chemical compositions, vapour, humidity, torque and others mechanical constraints [1–7]. Their most outstanding property is that they allow without a battery and wireless interrogation, as they are connected only by a radio frequency link to a transceiver or reader unit. Small size, light-weight, low cost, and maintenance-free are their other attractive features. All these SAW-based sensors work on the basis of measuring changes in the delay time or velocity of a surface wave due to the impact of a physical quantity being measured on the SAW propagation. For vibration sensing utilizing SAW technology, Filipiak *et al.* present some feasibility studies on such issue [8–10]. A prototype of SAW based vibration sensor with beam structure was developed for electronic warning system, and it was composed of a piezoelectric ST-cut quartz cantilever beam with a SAW delay line pattern fabricated on top of the beam surface and an aggregated mass attached on undamped end of the beam. The vibration directed the aggregated mass changes both the distance between the transducers and the stresses in the region where the surface wave propagates. The vibration is evaluated by measurements of changes in the delay time of SAW. A simple isotropic model with one degree of freedom was used to describe theoretically the sensor plate movement and identification of the vibrating sensor. Measurement range of 10 g, frequency range up to 20 Hz, and acceleration sensitivity of 8.5 kHz/g were obtained in the experimental results [8]. However, the present SAW vibration sensor still suffers from the poor temperature compensation, also, the active sensor structure makes the sensor is difficult for wireless vibration measurement in some extreme environments because of the power system. Additionally, the lack of the theoretical analysis on response mechanism inhibits the optimization of the sensor

**Figure 1.**
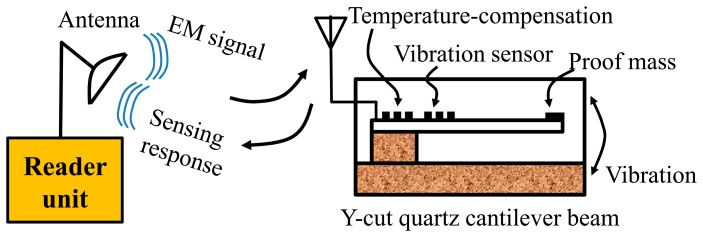
The schematic and principle of the wireless surface acoustic wave (SAW) vibration sensor.

Operating the similar principle, a novel wireless and passive temperature-compensated vibration sensor based on SAW technology was present in this study for on-line vibration monitoring of larger mechanical equipment as reciprocating compressor. The schematic of the presented sensor is depicted in Figure 1, it is composed of a flexible piezoelectric Y-cut quartz cantilever beam with a relatively substantial proof mass at the undamped end, and two one-port SAW resonators deposited directly on surface of the beam by using the photolithographic technique. One resonator acts as the sensing device adjacent to the clamped end for maximum strain sensitivity, and the other one is used as the reference located on clamped end for temperature compensation through the differential approach [11]. Y-cut quartz is chosen as the piezoelectric material for the cantilever owing to its linear temperature characteristics and good strain sensitivity [12]. When the interdigital transducers (IDTs) of the resonators receive electromagnetic (EM) energy from reader unit through antennas, the SAW is generated and reflected by reflectors due to the piezoelectric effect. The reflected wave is reconverted into EM waves by the IDTs and transmitted to the reader unit. Vibration directed to the proof mass flex the cantilever, inducing relative changes in the acoustic propagation characteristics of the SAW travelling along the sensing device, and generated output signal varies in frequency as a function of vibration. The sensor signal is demodulated wirelessly by evaluating the differential frequency shifts of the reflected impulses utilizing the reader unit. This sensor presents many advantages over other currently available vibration sensors: (1) it provides high sensitivity through optimal design by using the established theory model on response mechanism; (2) the temperature dependence is compensated effectively by using the differential structure; (3) the sensor chip is absolutely passive and does not require a battery or any power supply to operate; and (4) it is light, small, and can withstand extremely harsh environmental conditions.

To determine the optimal dimensions of the cantilever beam, a theoretical modelization is proposed by analyzing the strain distribution along the piezoelectric cantilever beam [7,13], which allows to obtain analytical expressions of the acceleration sensitivity. Accordingly, the dimension of the cantilever beam is determined for best strain sensitivity. Also, coupling of modes (COM) model, an efficient technique for SAW device simulation [14–16], was used to find optimal design parameters of the SAW devices prior to fabrication. Using the precise vibration table, and programmable incubator chamber, the performance of the developed SAW based vibration sensor was evaluated in wireless measurement referring to the interrogation unit.

## 2. Theoretical Analysis

### 2.1. Vibration Response Mechanism

**Figure 2.**
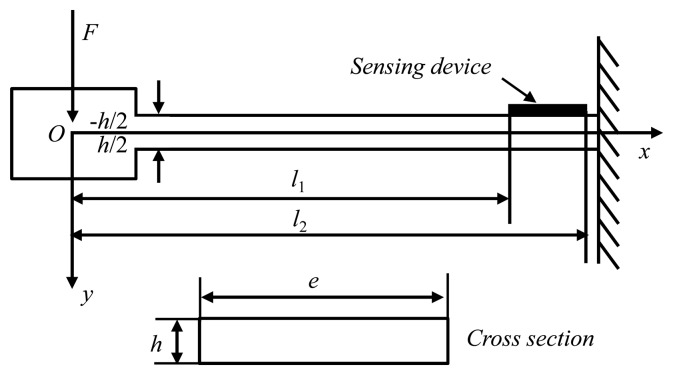
The static model of the vibration sensor with cantilever beam.

In this paper, we propose a theoretical approach for analyzing vibration response mechanism of SAW vibration sensor composed of a Y-cut quartz cantilever beam with a relatively substantial proof mass at the undamped end, and a SAW device deposited directly adjacent to the clamped end surface of the beam as shown in Figure 2. The length, width, and thickness of the cantilever beam are indicated as *l*, *e*, and *h*, respectively. Also, to simplify the numerical solution, the ST-X quartz beam is assumed as isotropic medium. The inertial force *F* (*F* = *ma*, *m* and *a* are the proof mass and applied acceleration respectively) from the vibration directed to the proof mass, and induces shifts in stress σ*_Ax_* and strain (longitudinal in *x*-direction and transverse in *y*-direction: *ε_x_* and *ε_y_*) along the beam, which are expressed as [16].

(1) $$\begin{array}{l} \sigma_{Ax}=\frac{F\cdot x\cdot \left(-h/2\right)}{J}=-\frac{F\cdot x\cdot h}{2J}\\ {ɛ}_{x}=\frac{F}{E\cdot J}\cdot \frac{h}{2}\cdot x,{ɛ}_{y}=-\mu \frac{F}{E\cdot J}\cdot \frac{h}{2}\cdot x\\ J=eh^{3}/12 \end{array}\left(0<x<l\right)$$

here, *μ* and *E* are the Poisson's ratio and elastic modulus of the quartz cantilever. *J* is the rotational inertia. So the average strain distribution (*ε̄_x_* in *x*-direction and *ε̄_y_* in *y*-direction) in the effective resonance cavity of the SAW resonator on the clamped end of the cantilever is given by

(2) $$\begin{array}{l} {\bar{ɛ}}_{x}=\frac{\int_{l_{1}}^{l_{2}}\left(\frac{F}{E\cdot J}\cdot \frac{h}{2}x\right)dx}{l_{2}-l_{1}}=\frac{F}{E\cdot J}\cdot \frac{h}{2}\left(l_{2}+l_{1}\right)\\ {\bar{ɛ}}_{y}=-\mu \frac{F}{E\cdot J}\cdot \frac{h}{4}\left(l_{2}+l_{1}\right) \end{array}$$

For the vibration sensing, the inertial force *F* directed to the piezoelectric cantilever, the induced bending changes the center distance of interdigital electrodes. Additionally, the shift in strain along the cantilever beam induces the change of the density and elastic modulus of the beam, and also the shifts of the SAW velocity and resonance frequency. The resonance frequency shift, Δ*f*, depending on changes in strain induced by the vibration can be expressed as

(3) $$\Delta f=\frac{\left(r_{1}-1\right)\bar{{ɛ}_{1}}+r_{2}\bar{{ɛ}_{2}}}{1+\bar{{ɛ}_{1}}}f_{0}$$

here, *ɛ̄*_1_ and *ɛ̄*_2_ are the average flexing strain parallel and perpendicular to the surface acoustic wave propagation direction, respectively. *r*_1_ and *r*_2_ are the strain coefficients. *f*_0_ is the undisturbed resonance frequency. The *ɛ̄*_1_ in the denominator of Equation (3) can be ignored when ε < 10^−3^, so, the Equation (3) can be rewrite as

(4) $$\Delta f=\left(\left(r_{1}-1\right)\bar{{ɛ}_{1}}+r_{2}\bar{{ɛ}_{2}}\right)f_{0}$$

then, substituting *ɛ̄*_1_ = *ɛ̄_x_*,*ɛ̄*_2_ = *ɛ̄_y_* into Equation (4), the relationship between the resonance frequency response and applied acceleration *a* can be described as

(5) $$\Delta f=\frac{3ma}{Eeh^{2}}\left(l_{2}+l_{1}\right)\left(\left(r_{1}-1\right)-\mu r_{2}\right)f_{0}$$

### 2.2. Numerical Results and Discussion

**Figure 3.**
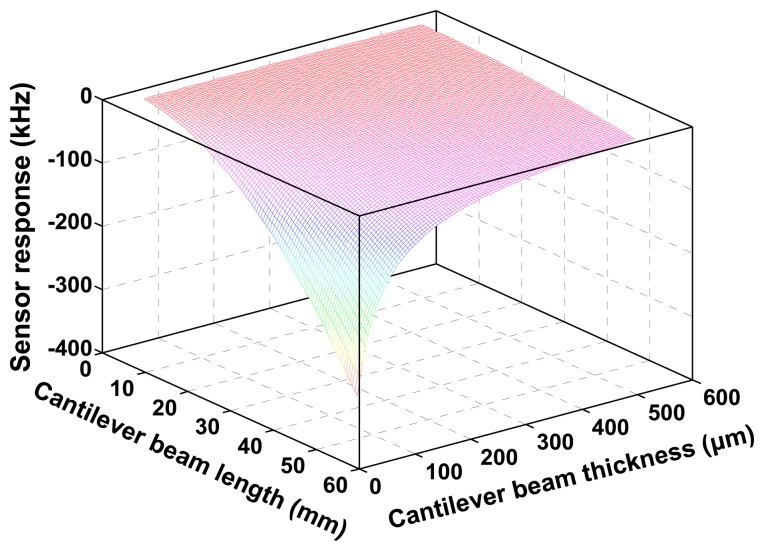
The calculated relationship among the sensor response, length and thickness of the cantilever beam, applied acceleration: 1 g.

The relationship among the sensor response and the dimension of the cantilever beam was calculated as shown in Figure 3 using Equation (5) and simulation parameters listed in Table 1. From the picture, the sensor response relates to the geometric structure of cantilever beam and proof mass. Larger mass loading, longer and thinner beam will significantly contribute the sensitivity. However, to avoid the resonance phenomena between the dynamic load and the cantilever beam, higher resonance frequency of the beam itself is essential, and it is given by the Rayleigh method using determined Youngs modulus [8]. In this study, the beam resonance frequency is assumed to over 500 Hz, and hence, the length of Y-cut quartz beam with 0.2 mm should be less than 20 mm according to the analytical expressions of the beam resonance frequency mentioned in Ref. [9]. Notably, the width of the cantilever does not significantly contribute to the sensor response and resonance frequency as mentioned in Equation (5), so, the width of the beam is considered as a constant, and it is set to 1.5 mm in our study. Consequently, the geometric of the cantilever is determined as 1.5 mm × 20 mm × 0.2 mm, and the proof mass and the beam mass ratio is set to 5. Based on the determined design parameters, the frequency sensitivity to acceleration from the vibration can be illustrated as shown in Figure 7c, expected sensitivity is around 11 kHz/g.

## 3. Coupling of Modes (Com) Simulation on Saw Devices

It is well known that the (COM) model is an efficient technique for SAW device simulation [14,15], and it was used to determine the design parameters of the one-port SAW resonator prior to fabrication. The aim is to obtain low insertion loss and high quality value. For simulation on the one-port resonator configuration composed of one IDT and two adjacent shorted reflectors as shown in Figure 1a, the COM model was used to analyze the IDT and reflectors reflectively. By using the cascading mixed P-matrix of the IDT, reflectors, and cavity between the IDT and reflectors [13], the frequency response S_11_ and S_12_ are obtained as

(6) $$\begin{array}{l} S_{11}=20\times \log\left(\left(R\times P_{33}-1\right)/\left(R\times P_{33}-1\right)\right)\\ ul=4\times abs{\left(R/\left(2R+1/P_{33}\right)\right)}^{2}\\ S_{12}=10\times \log\left(ul\right) \end{array}$$

where, *R* is the match impedance of the input and output port, and the real part and image part of the cascading P-matrix element, *P*_33_, represents the admittance of one-port resonator.

**Figure 4.**
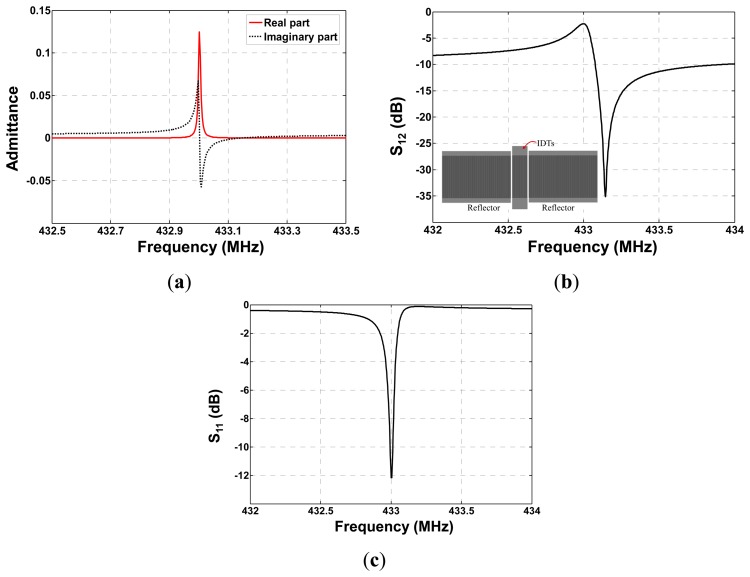
Coupling of Modes (COM) simulation on one-port SAW resonator, (**a**) admittance; (**b**) insertion loss (S_21_); and (**c**) reflection coefficient S_11_.

According to Equation (6), the admittance, frequency response S_11_ and S_12_ of the SAW resonator were simulated as shown in Figure 4. Y-cut quartz provides SAW velocity of 3159 m/s and electromechanical coupling constants of 0.12%. The operation frequency of the resonators is set to operate at 433 MHz. Number of IDT finger pairs and reflector electrodes with 1000 Å aluminum metallization of two resonators are set to 60 and 340, the finger widths are 1.823 μm, respectively. The cavity between the IDT and adjacent reflectors are both 0.625 λ (λ: wavelength at operation frequency). Larger Q-value over 14,000 and low insertion loss less than 3 dB were observed from the simulated results.

## 4. Technical Realization

**Figure 5.**
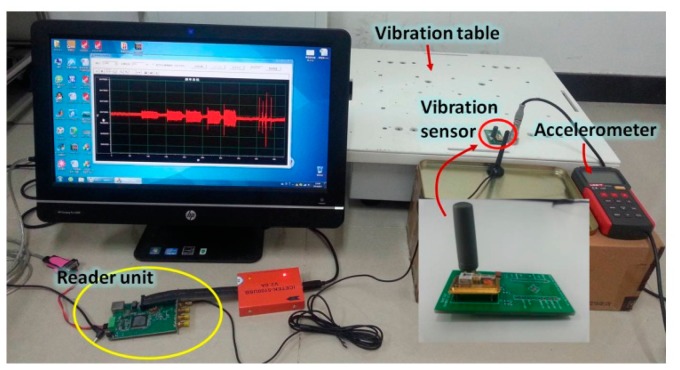
The wireless measurement setup of the SAW vibration sensor.

**Figure 6.**
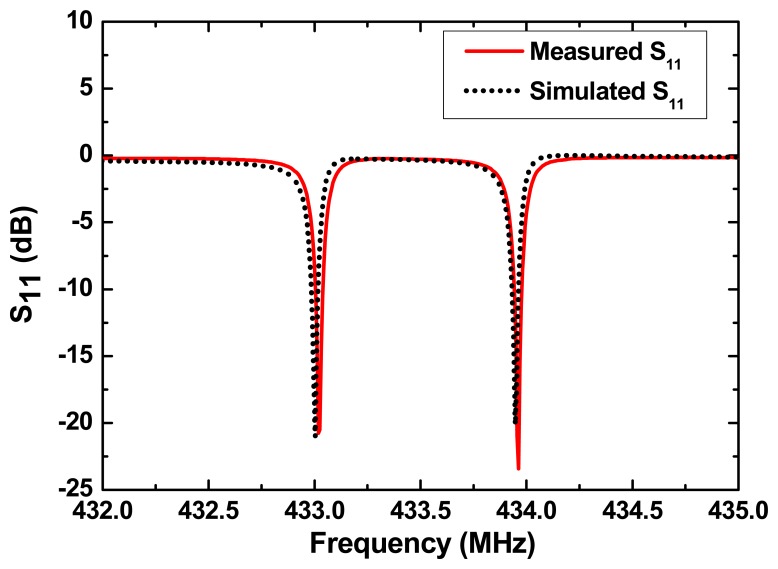
Simulated and measured S_11_ of the SAW resonator.

Based on the extracted optimal design parameters for the SAW resonator and cantilver beam, the chip for vibration sensor was reproducibly fabricated on the Y-cut quartz substrate by standard photolithographic technique, on which 1000 Å aluminum IDTs and adjacent shorted grating reflectors were deposited. The operation frequencies of the resonators are designed to operate at 433 MHz and 434 MHz, respectively, which provides a differential frequency of 1 MHz for vibration sensing. Number of IDT finger pairs and reflector electrodes of two resonators are both set to 60 and 340, the finger widths are 1.823 μm and 1.819 μm, respectively. The cavities between the IDT and adjacent reflectors are both 0.625 λ (λ: wavelength at operation frequency), which provide lower insertion loss and higher unloaded quality (Q) value. The dimensions of the Y-cut quartz beam are set to 1.5 mm × 20 mm × 0.2 mm depending on the theoretical optimum. Then, the Y-cut quartz cantilever beam with two one-port SAW resonator patterns were clamped by using the fixed base made by Al. The proof mass with five times the mass of cantilever beam is affixed at the undamped end. Next, the fixed base was affixed firmly onto the metal package base. The developed packaged vibration sensor with a dipole antenna connected is depicted in Figure 5. The typical electrical resonance characteristics of the developed one-port SAW resonator are measured by using the Agilent E5071B Network analyzer, obtained unloaded Q-value is up to 12,000. The measured operation frequencies of two resonators are 433.02 MHz and 433.95 MHz, respectively. All measured S_11_ were well matched with predicted values from the COM simulation, as shown in Figure 6.

For wireless measurement, an interrogation unit (reader) utilizing the “frequency domain” method was employed (as shown in Figure 6), which interrogates a specific frequency with narrow-band pulse and then measures the returned signal power [17].

## 5. Experimental Results and Discussions

Then, the developed vibration sensor system is characterized wirelessly. The measurement setup consists of the developed SAW vibration sensor, reader unit, precision vibration table, incubator chamber, and commercial accelerometer for calibration as shown in Figure 6. The RF power from the reader unit is set to 20 dBm and the maximum readout distance of ∼1.5 m is observed. The precision vibration table provides acceleration range of 0∼10 g with varying vibration frequency and the applied vibration is monitored by using the commercial accelerometer. The SAW vibration sensor was bolted firmly to the vibration table. A self-made interface display program was use to real-time record and display the differential frequency signal.

**Figure 7.**
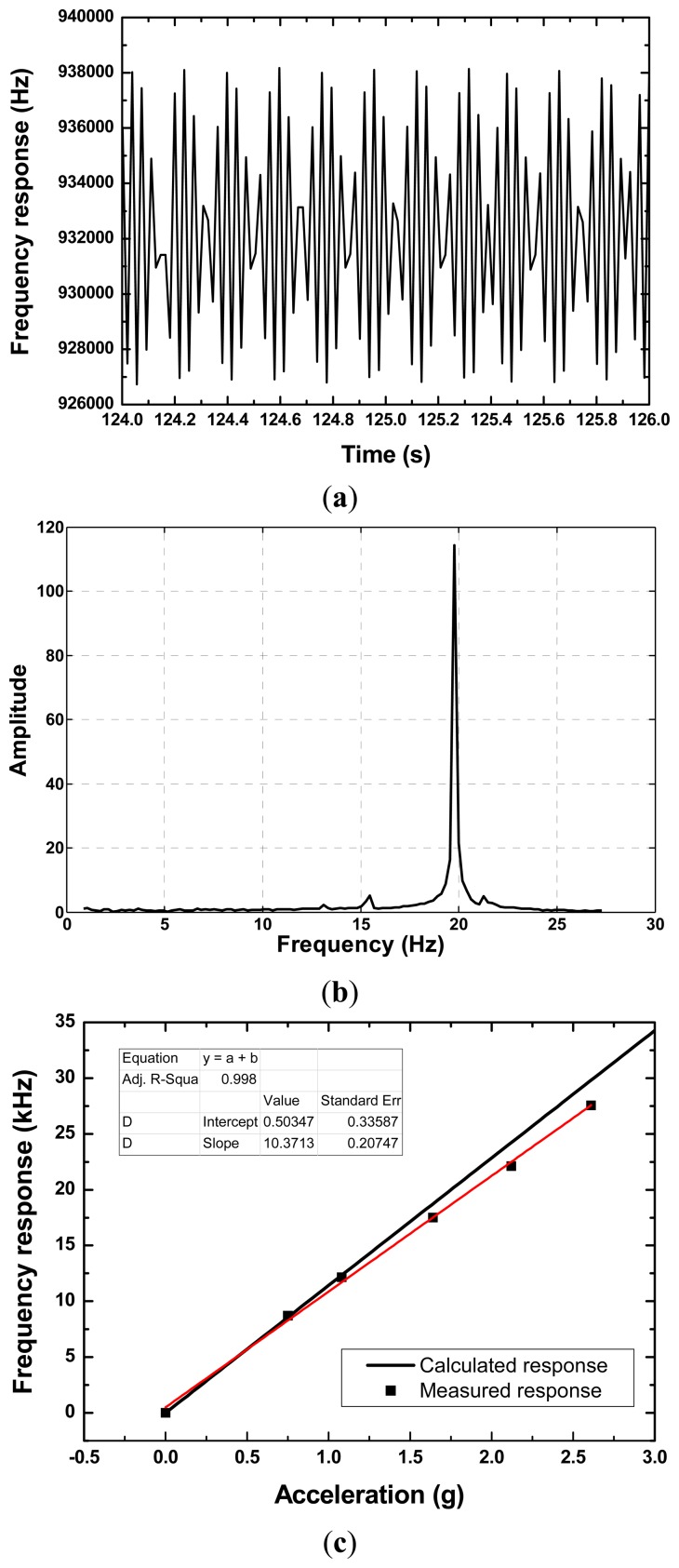
(**a**) Measured transient response; (**b**) spectrum; and (**c**) vibration sensitivity of the developed vibration sensor, and also in comparison to the calculated result.

**Figure 8.**
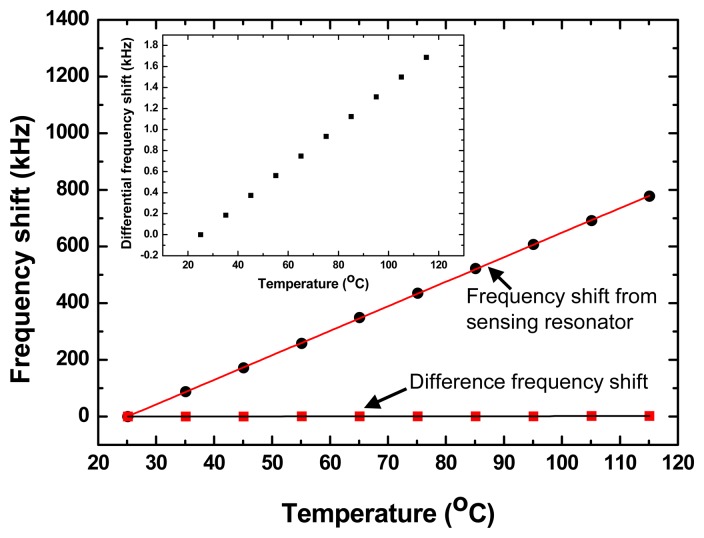
Measured frequency-dependence from the sensing resonator and differential structure, inset: an expansion of the difference frequency.

Figure 7a,b shows the continuous response and the spectrum of the stimulated sensor at a homogenous vibration with acceleration of 1.08 g and testing temperature of 25 °C. The frequency response t vibration was recorded every 18 ms, so that one point on the graph corresponds to an 18 ms interval. The spectrum in Figure 7b indicates the vibration frequency is ∼20 Hz, and it is obtained on FFT analysis of the continuous response in Figure 7a, and it consistent well with the settings of the vibration table. The magnitude of the recoding signals is proportional to the acceleration, as shown in Figure 7c. The measured results indicate that high sensitivity of ∼10.4 kHz/g and good linearity was obtained. Also, good agreement was observed among the measured sensitivity and calculated result. Additionally, the cross-temperature sensitivity of the vibration sensor was also characterized by using the incubator chamber, the difference frequency-dependence is only 18.6 Hz/°C in testing temperature range of 20∼120, far less than the cross-temperature sensitivity of the single sensing resonator (8.6 kHz/°C), as shown in Figure 8. It means good temperature compensation can be obtained in the vibration sensing by using the differential structure. From the results, we suggest that this prototype SAW vibration sensor is very promising for achieving wirelessly requestable and batteryless on-line vibration sensing application on the larger mechanical equipment.

## 6. Conclusions

A novel wireless and passive SAW based temperature-compensated vibration sensor incorporating a Y-cut quartz cantilever beam was successfully demonstrated. A theoretical analysis was performed to determine the optimal dimension of the cantilever beam. The SAW device used for the sensor was simulated by using the COM model prior to fabrication. The developed vibration sensor was wirelessly characterized by using the precision vibration table. High sensitivity of ∼10.4 kHz/g, excellent linearity, and good temperature compensation were obtained for vibration sensing.

## Figures and Tables

**Table 1.** Simulation parameter of the SAW vibration sensor.

	*r*_**1**_	*r*_**2**_	*E***/***N***·***m*^**−2**^	*μ*	*ρ***/***kg***·***m*^**−3**^	*f*_**0**_**/MHz**
Y-cut quartz	+ 0.439 ± 0.030	+ 1.319 ± 0.064	7.83 × 10^10^	0.14	2631	434
